# Effects of *Trichoderma* seedling treatment with System of Rice Intensification management and with conventional management of transplanted rice

**DOI:** 10.7717/peerj.5877

**Published:** 2019-01-11

**Authors:** Ram B. Khadka, Norman Uphoff

**Affiliations:** 1 Regional Agricultural Research Station, Nepal Agricultural Research Council, Khajura, Banke, Nepal; 2 Department of Plant Pathology, The Ohio State University, Wooster, OH, United States of America; 3 SRI-Rice, International Programs (IP/CALS), Cornell University, Ithaca, NY, United States of America

**Keywords:** Beneficial Microbes, Premium Rice Land-races, Organic Farming

## Abstract

Many benefits of *Trichoderma* inoculation for improving crop production have been documented, including growth and yield enhancement and the alleviation of biotic and abiotic stresses. However, because rice is usually cultivated under continuous flooding that creates anaerobic soil conditions, this limits the benefits of these beneficial fungi. Cultivating rice with the methods of the System of Rice Intensification (SRI) provides rice plants with a more favorable environment for their colonization by beneficial microbes in the soil because the soil is more aerobic under SRI management and contains more organic matter. This study evaluated the effects of *Trichoderma* inoculation of rice plants under SRI management compared with transplanted and flooded rice plants, considering also the effects of different means of fertilization and different varieties in rice. Experiments were conducted in 2015 and 2016 under the tropical climate of Nepal’s western terai (plains) during both the rainy season (July to November) and the dry season (March to July). The results indicated significantly better performance (*P* = 0.01) associated with *Trichoderma* inoculation for both seasons and for both systems of crop management in terms of grain yield and other growth-contributing factors, compared to non-inoculated rice cropping. Relatively higher effects on grain yield were recorded also with organic compared to inorganic fertilization; for unimproved (heirloom) varieties compared with improved varieties; and from SRI vs. conventional flooded crop management. The yield increase with *Trichoderma* treatments across all trials was 31% higher than in untreated plots (4.9 vs 4.5 mt ha^−1^). With *Trichoderma* treatment, yields compared with non-treated plots were 24% higher with organic SRI (6.38 vs 5.13 mt ha^−1^) and 52% higher with non-organic SRI (6.38 vs 3.53 mt ha^−1^). With regard to varietal differences, under SRI management *Trichoderma* inoculation of the improved variety Sukhadhan-3 led to 26% higher yield (6.35 vs 5.04 mt ha^−1^), and with the heirloom variety Tilkidhan, yield was 41% higher (6.29 vs 4.45 mt ha^−1^). Economic analysis indicated that expanding the organic cultivation of local landraces under SRI management should be profitable for farmers where such rice has a good market price due to its premium quality and high demand and when SRI enhances yield. These varieties’ present low yields can be significantly increased by integrating *Trichoderma* bio-inoculation with SRI cultural methods. Other recent research has shown that such inoculation can be managed profitably by farmers themselves.

## Introduction

Microorganisms are a vital but unseen constituent of farming systems. Many free-living microorganisms establish beneficial biological relationships with crop plants by living in the rhizosphere around the roots and by colonizing plants’ roots, an effect not limited to leguminous crops. Some microorganisms also inhabit tissues and cells within plants’ above-ground organs as well as in their roots, living inside the plants as symbiotic endophytes (Uphoff et al., 2013).

While many microorganisms are predatory or parasitic, the majority live harmoniously and even beneficially with their plant hosts (Parmar & Dadarwal, 1999). They have capability to influence plants’ physiological processes, enhance their growth and development, and make plants more tolerant of biotic and abiotic stresses (Dardanelli et al., 2010; Berendsen, Pieterse & Bakker, 2012).

The concept of *microbiota*, which refers to the ensemble of diverse microbes, particularly bacterial and fungal, that reside around, on, and inside plants (the latter as endophytes), has been gaining currency in the literature (Müller et al., 2016; Hacquard, 2016). So has the concept of *holobionts*, which refers to the composite entities where microbiota are living symbiotically with their host plants (Margulis & Fester, 1991).

To date, most attention has been given to free-living endophytic bacteria such as plant growth-promoting rhizobacteria (PGPR) (Glick, Karaturovíc & Newell , 1995). But free-living beneficial fungi known as plant growth-promoting fungi (PGPF) may be as significant. Much research has already been done on mycorrhizal fungi, which are resident in plants’ roots and acquire significant amounts of water and nutrients (especially P) through their hyphal filaments which extend from the root system into the surrounding soil. These symbionts share the water and nutrients with their plant cell hosts and in turn receive carbohydrates and other compounds that have been synthesized by the plant (Hirsch et al., 2003; Sarma, Oehrle & Emerich, 2007; Parniske, 2008; Lanfranco, Fiorilli & Gutjahr, 2018).

The study of mycorrhizal fungi extends back well over 150 years (Frank, 2005). In more recent years, increasing attention has been given to other fungi that have symbiotic, mutually-beneficial relationships with plants. These fungi can enhance crops’ growth and yield through the production of phytohormones, through phosphate solubilization, through cellulose degradation, and siderophore production (Smith & Read, 2008; Balestrini et al., 2012; Doni et al., 2017).

These fungal microbes change the physiochemical properties of the rhizosphere, among other things producing exopolysaccharides and forming biofilms. They also induce osmoprotectants and heat-shock proteins in cells, thereby making the plant better able to resist abiotic stresses such as salinity, drought, cold, and high temperature (Redman et al., 2002; Rodriguez et al., 2009; Redman et al., 2011; Grover et al., 2011). Furthermore, plant-fungi associations can change the growing environment, making it more congenial for plants’ growth and development (Schenk, Carvalhais & Kazan, 2012). Thus, free-living PGPF play a vital although unobserved role in terrestrial ecosystems (Cox, Eaton & Scott, 2010). Here we focus on a less-studied fungal genus that can have similar beneficial effects.

*Trichoderma* (teleomorph *Hypocrea*) is a ubiquitously-distributed genus of fungi that can be symbiotically associated with plant roots (Vargas, Mandawe & Kenerley, 2009). These fungal species provide major benefits in farming systems such as the mitigation of biotic and abiotic stresses (Harman, 2000; De Souza et al., 2008; Harman et al., 2010); the enhancement of plant growth regulators (Yedidia et al., 2001; Harman & Shoresh, 2007; Shoresh, Harman & Mastouri, 2010); bioremediation and detoxification of harmful chemicals such as DDT, dieldrin, endosulfan, pentachloronitrobenzene, and pentachlorophenol (Katayama & Matsumura, 1993); suppression of phytopathogens; nutrient mobilization in the rhizosphere; and enhancement of plants’ defense mechanisms (Bae et al., 2009).

Higher biomass production, better seedling vigor, and lower impact of biotic and abiotic stresses have been observed in a variety of crops when their seeds were treated with *Trichoderma* (Chang, 1986; Mastouri, Björkman & Harman, 2010; Rego et al., 2014)*.* However, because rice, the staple food for over half the world’s population, is grown in inundated fields wherever there is sufficient water available, this flooding creates a soil environment that is inhospitable for *Trichoderma* and other fungi because these aerobic organisms cannot grow well under hypoxic flooded conditions.

A rice culture methodology that is gaining attention and acceptance, known as the System of Rice Intensification (SRI), relies on alternate wetting and drying (AWD) of rice paddies rather than on their continuous inundation. AWD enhances water use efficiency in rice production along with increased land and water productivity (Carrijo, Lundy & Linquist, 2017). This change in standard water management methods for growing rice creates a hospitable environment for aerobic soil microbes, both bacteria and fungi. SRI methods enhance the soil environment for microbes by favoring the use of organic materials for soil fertilization in preference to inorganic means. Further, the use of a simple mechanical rotary weeder to control weeds breaks up and aerates the top layers of soil at the same time that it eliminates weeds, burying them as green manure, which also enhances the environment for soil microbial populations (Anas et al., 2011).

SRI achieves higher productivity by improving the growing environment for rice rather than by relying on making changes in crop genetic potential and applying agrochemical inputs. While there are some benefits from continuous flooding of the soil as done in conventional rice production, it has deleterious effects for rice root systems, such as deforming their cortex to create air pockets (aerenchyma) in the roots (Kirk & Bouldin, 1991), inhibiting root respiration, and causing degeneration of roots due to hypoxia (Kar et al., 1974). Continuous flooding can slow down root metabolism, ion transport, and root and canopy growth (Barison & Uphoff, 2011) at the same time that it suppresses beneficial soil microbes which thrive under aerobic conditions.

By rapidly depleting the oxygen level in bulk soil (Liesack, Schnell & Revsbech, 2000), flooding promotes the growth of anaerobic microorganisms such as fermentative bacteria and methanogenic archaea so that these become dominant rather than PGPR and PGPF. The practices of SRI create a different and more productive environment for growing rice. They combine a number of changes in the standard methods of rice production: plant density is optimally low; very young seedlings are transplanted with minimum ‘shock’; paddy soils are alternately wetted and dried, not flooded; active soil aeration is part of weeding operations; and soil organic matter is enhanced (Thakur, Uphoff & Antony, 2010; Barison & Uphoff, 2011).

These practices suggest that SRI should be compatible with, and its benefits could be further amplified by, the incorporation of *Trichoderma* into this rice cropping system. One feature of SRI methodology is the transplanting of young seedlings, for better crop establishment, before the start of their 4th phyllochron of growth, so as to maintain more of the plants’ inherent potential for tillering and root growth (Nemoto, Morita & Baba, 1995). Transplanting rice seedlings from a nursery into the field, however, is an operation in which seedlings are vulnerable to injury, disease, and abiotic stresses. The treatment of seedlings with *Trichoderma* during transplanting operations could help young rice plants buffer these stresses, with a positive impact on eventual yield.

Research is needed on the possible role of *Trichoderma* spp. in enhancing rice crop phenology and yield, to assess the potentials of these fungi to act as plant growth regulators/promoters and as disease suppressors. This present study was designed to evaluate the effects that native *Trichoderma* isolates, taken from local soils in Nepal, might have on the growth and performance of rice plants grown under SRI management vs. conventional methods (transplanting of older seedlings, closely spaced, and flooded) and also to assess the effects of organic vs. inorganic fertilization as well as of using improved vs. local varieties under the agroecosystem conditions of western Nepal.

## Materials and Methods

Experiments were conducted in two consecutive seasons: a rainy season (July to November 2015), and a dry season (March to July 2016). Both experiments were conducted in a tropical environment, 181 m above sea level, at the Regional Agricultural Research Station (RARS) in Khajura, Banke district, Nepal. This is located at 81°37″E longitude and 28°06″N latitude. The soil of the experimental plot was sandy loam (68% sand, 22% silt, and 10% clay), poor in organic matter (1.97%) and available potassium (97.7 kg ha^−1^), and medium in available nitrogen (0.16%) and phosphorus (51.4 kg ha-1). The soil pH was neutral to slightly alkaline (7.2).

The first experiment was conducted with a split-plot design where the main plot evaluated the methods of SRI with either organic or inorganic fertilization. Two different varieties were planted in the subplots. A popular local unimproved variety known as Tilki (formally designated as Tilkidhan #8) was evaluated. Its seeds were obtained from a community seed bank in Beluwa, Bardia, which is managed by a non-governmental organization, Local Initiatives for Biodiversity, Research, and Development (LI-BIRD), based in Pokhara, Nepal. This ‘heirloom’ landrace which has a crop cycle of 145–150 days is considered to have medicinal properties and to be tolerant of flooding. Its market price is high because of its unique aroma and texture (Maharjan, Gurung & Sthapit, 2013). The improved variety used in the first trials was a drought-tolerant variety, Sukha-3 (Sukhadhan-3), which has medium-length grain and a relatively short cycle, 125 days. This recently-released, photoperiod-insensitive variety was originally introduced from the International Rice Research Institute (IRRI) in the Philippines.

For the SRI trials, 10-day-old seedlings were transplanted individually at spacing of 25 × 25 cm in a grid square pattern. Alternate drying and wetting was maintained as much as possible up to 60–70 days after transplanting (DAT). A rotary weeder was used at 25 and 45 DAT to remove weeds and create some aeration of the rhizosphere. In the inorganic treatments, soil fertilization was done with 100 kg of nitrogen, 30 kg of phosphorus, and 30 kg of potassium per hectare, provided respectively through urea, diammonium phosphate, and potassium chloride, along with 10 tonnes per ha of farmyard manure (FYM). In the organic treatments, 20 tonnes of farm yard manure (FYM) per ha were applied, without any amendments of inorganic fertilizer. With both methods of cultivation, the FYM was incorporated one week before transplanting. For the inorganic fertilization, 50% of the N and all of the P and K were applied at the final stage of land preparation (puddling), with the remaining 50% of N applied in split treatments at 30 and 60 days after transplanting.

The second experiment was conducted with a similar factorial design where the main factor was seedling treatment, either with *Trichoderma* (bio-inoculated) or no treatment (no inoculation), with then three variants of management as the sub-factors: organic SRI (org SRI), inorganic SRI (inorg SRI), and conventional transplanting (CT). In this experiment, a short-duration (125-day) variety (Chaite-2) was used, recommended for dry-season cultivation in the terai (southern plain) and inner terai (plain region between Churiya hills and Mahabharat mountain range) regions of Nepal. All of the agronomic practices in SRI method were applied as mentioned earlier; however, the age of seedling was 18 days (at 3-leaf stage) because of the slower seedling growth associated with lower night temperatures in that season. In the conventional management trials, seedlings were transplanted at the 5–6 leaf stage, with 15 × 15 cm spacing and three seedlings per hill. This made the respective plant densities 133 plants per m^2^ for CT and 16 plants per m^2^ in the SRI trials. The researchers tried to keep the CT plots continuously flooded during the crop’s entire life cycle, except from 15–20 days before harvesting.

In both sets of experiments, the individual plot sizes were 15 m^2^, and each treatment was replicated three times. For the bio-inoculation treatments, the seedlings were dipped in a *Trichoderma* spore suspension for 10 to 20 min before transplanting. The *Trichoderma* strain used was one reported to be a native isolate of Nepal, *Trichoderma viride* which is commercially produced and distributed by Agri-Care Nepal Pvt. Ltd. based in Bharatpur, Chitwan. The final concentration of the suspension used for seedling treatment was about 10^6^ spores per ml. In the non-inoculation treatments, seedlings were transplanted without such treatment. For weed control in the SRI trials, a rotary weeder was used two times at 20 and 40 days after transplanting, to also give aeration to the rhizosphere; in the CT trials, hand weeding was done at 30 days after transplanting.

Data were collected on grain yield, grain weight (thousand-grain weight in g), panicle length, number of panicles hill^−1^, number of panicles m^−2^, plant height, and number of grains panicle^−1^. All these parameters were recorded during harvesting. Panicle length, number of panicles hill^−1^, number of panicles m^−2^, and number of grains panicle^−1^ were recorded from the central area of the plot to avoid any border effect. Grain moisture was measured with a moisture meter (WILE Twister Grain Moisture Meter), and both grain weight and grain yield were adjusted to a 14% grain moisture level.

The normality of the data was tested by the Shapiro–Wilk test. Normally-distributed data were subjected to analysis of variance (ANOVA) testing using RStudio (R-3.2.5) (RStudio Support, 2018). When there was a significant difference between the treatment means, Fisher tests of least significant difference (LSD) were applied. Data that were not normally distributed were transformed to log before proceeding for ANOVA and the value of back transformed were presented. Multiple Factor Analysis was also done in RStudio using packages FactoMineR and factoextra (Le, Josse & Husson, 2008).

## Results

### Effect of *Trichoderma* seedling treatment with different rice cultivation methods

Measures of grain yield, grain test weight, and number of panicles hill^−1^ averaged across the three different methods of crop establishment showed significantly better crop performance with *Trichoderma* seedling treatment. The values of these three parameters comparing bio-inoculated vs. non-inoculated plants in their respective plots were, respectively, 5.9 vs. 4.7 tonnes ha^−1^ for yield; 20.6 vs. 19.5 grams for 1000-grain weight; and 11.8 vs. 10.7 for panicles hill^−1^. The yield increases recorded with *Trichoderma* treatment thus averaged 26% across the three methods of crop establishment compared to non-treated samples. Grain weight with inoculation was 5% higher, and panicles hill^−1^ were 9% more.

**Figure 1:**
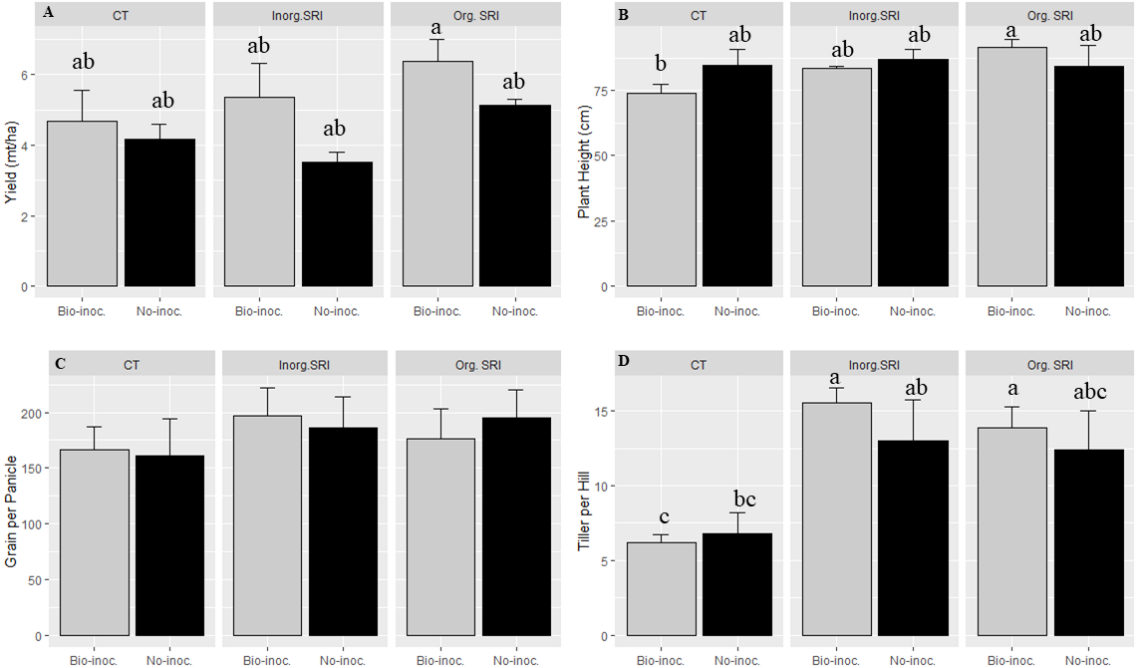
Growth and yield attributes affected by *Trichoderma* seedling inoculation and methods of crop establishment in dry season, 2016, Each data point indicates the average of three replications. The error bar indicates the standard error.

Similarly, there was significantly higher yield, grain weight, plant height, and more panicles m^−2^ in organic SRI that had been treated with *Trichoderma*. The average yield with *Trichoderma* inoculation and conventional cultivation methods was 12.5% higher compared to untreated plots. In comparison with results from non-treated rice plants using the respective methods in the dry season 2016, average yields from the *Trichoderma*-treated plants under organic SRI management were 24% higher, and they were 52% higher with inorganic SRI methods. We also observed significantly higher grain yield (*p* = 0.01; 6.1 vs. 4.8 mt ha^−1^), greater grain weight (21.9 vs 19.8 gm), and taller plant height (*P* = 0.02; 88.0 vs 85.2 cm) in the organic SRI compared to the inorganic SRI, as seen in Table 1 and Fig. 1.

**Table 1:** Growth and yield attributes affected by *Trichoderma* seedling inoculation and methods of crop establishment in dry season, 2016.

Treatments	Grain yield (mt ha^−1^)	Days to maturity	Plant height (cm)	Number of tillers per hill	Number of grains panicle^−1^	Test weight (gm)
**Inoculation**						
Bio-inoculation	5.9 ± 0.8a	135.1 ± 2.2	82.9 ± 8.0	11.8 ± 4.4	179.9 ± 24.7	20.6 ± 3.6
No inoculation	4.7 ± 1.1b	135.1 ± 2.0	85.3 ± 5.3	10.7 ± 3.5	180.8 ± 29.5	19.5 ± 1.9
*P*-value	<0.01	1.0	0.3	0.2	0.9	0.4
LSD	1.3	2.2	7.0	4.0	26.2	2.9
**Method of cultivation**						
Organic SRI	6.1 ± 1.14a	136.1 ± 2.5	88.04 ± 6.5a	13.1 ± 2.0a	186.1 ± 24.8	21.9 ± 3.9a
CT	5.1 ± 0.74ab	133.8+1.1	79.2 ± 7.4b	6.5 ± 1.0b	163.6 ± 25.1	18.4 ± 0.5b
Inorganic SRI	4.8 ± 1.2b	135.3 ± 1.8	85.2 ± 3.2ab	14.3 ± 2.3a	191.4 ± 24.5	19.8 ± 2.2ab
*P*-value	<0.01	0.2	<0.01	<0.001	0.2	0.1
LSD	1.3	2.5	7.5	1.9	28.9	3.1

**Notes.**

Means followed by same letter(s) do not differ significantly at 5% level of probability, *P*, Probability value, LSD, Least significant difference. CT, Conventional transplanting with chemical fertilizer: 100:30:30 kg NPK and 10 mt ha^−1^ of farmyard manure (FYM), Org SRI, Organic SRI with 20 mt ha^−1^ of FYM and no chemical fertilizer applied; in the case of SRI chemical fertilizer treatments, only 100:30:30 kg NPK were applied. Bio-inoc: Rice seedling treated with *Trichoderma* spore suspension before transplanting, Non-inoc: No inoculation with *Trichoderma*. The values behind ± indicates standard deviation.

### Effects of *Trichoderma* seedling treatment in heirloom vs. improved varieties with either organic vs. inorganic versions of SRI management

An experiment was conducted in the main rice-growing season 2015 at RARS in Khajura where two varieties of rice were planted with SRI methods—Tilki, an aromatic heirloom variety, and Sukhadhan-3, an improved, drought-tolerant variety—having either *Trichoderma* seedling treatment or no treatment. Different parameters were considered to understand the effects of variety, bio-inoculation, and fertilization, and their interaction, under SRI management.

**Figure 2:**
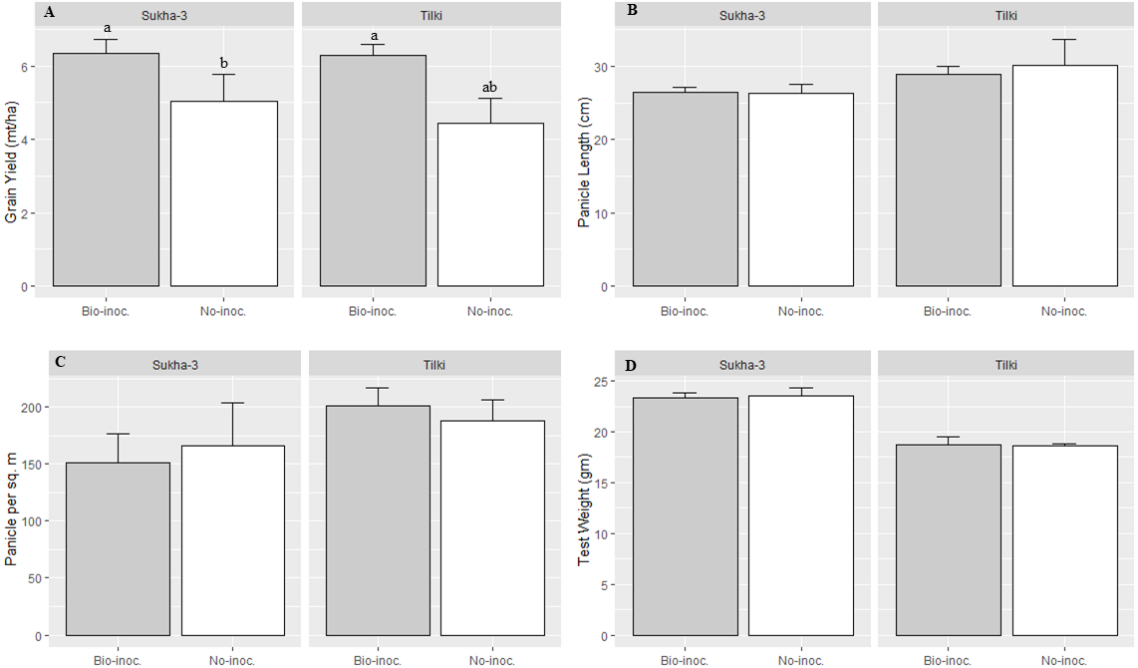
Effects of *Trichoderma* seedling treatment on yield and different growth attributes under SRI management in main season 2015. Each data point indicates the average of three replications. The error bar indicates the standard error. Means followed by same letter(s) do not differ significantly at 5% level of probability.

Analysis of variance for various crop phenological parameters showed significant differences among the treatments with *Trichoderma* inoculation or no inoculation, organic or inorganic fertilization, local or improved varieties, and their interactions. Significant effect on grain yield was observed across all the trials between organic and inorganic fertilization (*p* = 0.02). Furthermore, SRI management with organic fertilization, the values for number of panicles m^−2^, number of filled grains panicle^−1^, and panicle length were all higher compared to inorganic fertilization under SRI management (Table 2 and Fig. 2). Under SRI management, there was significantly higher grain yield (*p* < 0.001) on average across all trials in the *Trichoderma*-inoculated plots compared to non-inoculated rice plots.

**Table 2:** Effects of *Trichoderma* seedling treatment on yield and different growth attributes under SRI management in main season, 2015.

Treatments	Grain yield (mt ha^−1^)^z^	Panicle length (cm)	No. of filled grains panicle^−1^	No. of unfilled grains panicle^−1^	No. of panicles m^−2^	Thousand- grain weight (gm)
**Variety**						
Sukhadhan-3	5.6 ± 1.5 (0.7)	26.3 ± 0.9b	146.1 ± 20.2b	19.3 ± 3.4b	158.5 ± 31.7b	23.4 ± 0.6a
Tilkidhan	5.4 ± 1.5 (0.6)	29.5 ± 2.5a	219.3 ± 30.2a	40.5 ± 8.0a	194.5 ± 17.5a	18.6 ± 0.54b
*P*-value	0.4	<0.001	<0.001	<0.001	<0.001	<0.001
LSD	1.3	1.5	21.1	5.3	19.6	0.51
**Method of fertilization**						
Inorganic	5.0 ± 1.8 (0.6b)	27.2 ± 1.6	179.1 ± 46.8	29.0 ± 12.8	175.0 ± 39.0	21.09 ± 2.7
Organic	5.9 ± 0.9 (0.7a)	28.6 ± 3.0	186.3 ± 45.0	30.9 ± 12.4	178.0 ± 22.1	21.01 ± 2.3
*P*-value	0.02	0.08	0.42	0.32	0.8	0.2
LSD	1.2	2.06	40.01	11.19	25.8	0.71
**Inoculation**						
Bio-inoculation	6.3 ± 0.8 (0.8a)	27.7 ± 1.5	187.9 ± 48.0	29.6 ± 11.1	175.9 ± 3.9	21.01 ± 2.46
No inoculation	4.7 ± 1.6 (0.6b)	28.2 ± 3.2	177.5 ± 43.3	30.26 ± 14.1	177.25 ± 30.6	21.08 ± 2.45
*P*-value	<0.001	0.5	0.33	0.80	0.91	0.77
LSD	1.3	2.1	39.8	11.2	19.6	0.51

**Notes.**

Means followed by same letter(s) do not differ significantly at 5% level of probability, ns = non-significant at 5% level of probability. Bio-inoculation is seedling treated with *Trichoderma* spore suspension before transplanting, the values behind ± indicates standard deviation, ^z^ values inside the parenthesis indicates the log transformed values.

**Figure 3:**
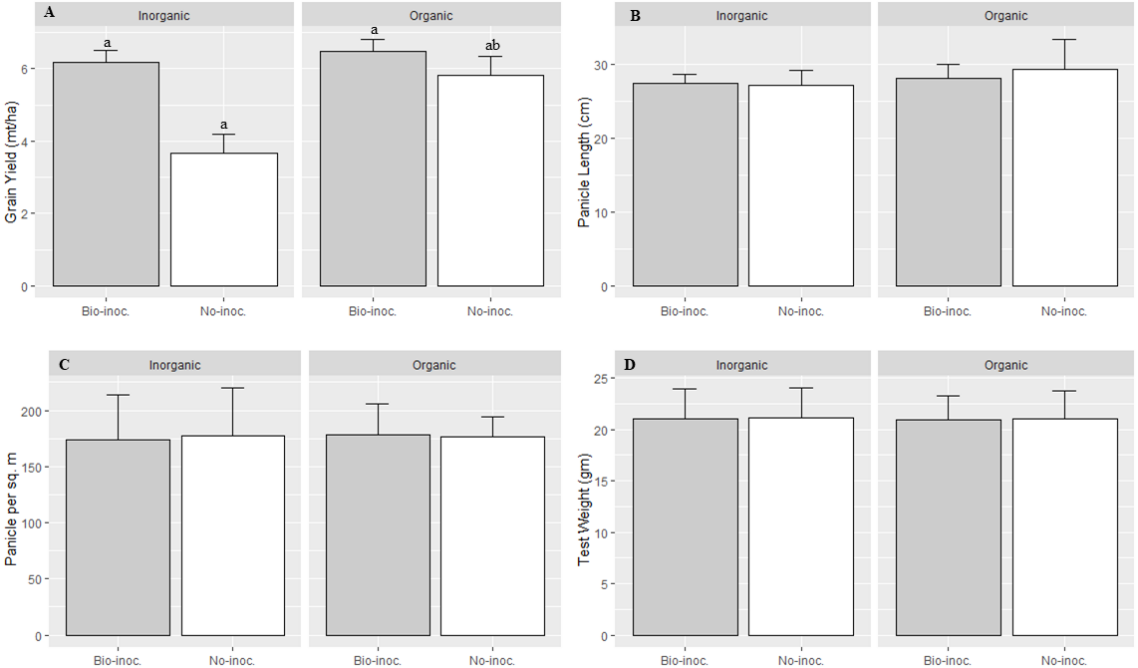
Effects of *Trichoderma* seedling treatment on yield and different growth attributes, average for two varieties, under SRI management in the main season, 2015. Each data point indicates the average of three replications. Means followed by same letter (s) do not differ significantly at 5% level of probability. The error bar indicate standard error.

Interaction effects of fertilization and bio-inoculation were found to be significant in grain yield, while other parameters were statistically at par in bio-inoculated and non-inoculated plots with organic and inorganic fertilization. The effect of *Trichoderma* inoculation on grain yield was significantly higher with organic compared to inorganic fertilization (Table 2 and Fig. 2). Further, the heirloom variety was found to be more responsive to bio-inoculation with *Trichoderma* than was the improved variety.

The heirloom variety Tilki had significantly longer panicle length and a higher number of filled grains than the improved variety Sukhadhan-3. However, no significant effect of bio-inoculation and fertilization was recorded in panicle length and number of filled grains panicle^−1^. The number of filled grains and number of panicles m^−2^ were higher in the bio-inoculated treatments than in the non-inoculated treatments (Table 2 and Fig. 3).

### Interaction effects of bio-inoculation, method of cultivation, and varieties on yield and yield-contributing parameters under SRI management

Significant effects of treatment interaction on grain yield were observed in both experiments. With SRI as the method of cultivation, the highest grain yield among all the treatment interactions was with Sukha-3 + bio-inoculation + organic fertilization (6.5 mt ha^−1^). This was, however, not much higher than the Tilki + bio-inoculation + inorganic fertilization treatment (6.3 mt ha^−1^). The lowest yield was with Tilki + non-inoculation + inorganic fertilizer, which showed that this landrace did not respond very favorably to chemical fertilizer and *Trichoderma* inoculation (Table 3).

**Table 3:** Effects of *Trichoderma* inoculation under SRI management in main rice season 2015.

Treatments	Grain yield (mt ha^−1^)^z^	Panicle length (cm)	Filled grains panicle^−1^ (no.)	Unfilled grains panicle^−1^ (no.)	Panicle m^−2^ (no.)	Test grain weight (gm)
Sukha-3 + bio-inoc + inorg	6.1 ± 1.2 (0.7ab)	26.3 ± 0.2	144.7 ± 17.8bc	22.3 ± 3.5abc	147.3 ± 36.6	23.5 ± 0.50a
Sukha-3 + bio-inoc + org	6.5 ± 0.5 (0.8a)	26.5 ± 1.0	158.8 ± 12.6bc	18.1 ± 2.6c	154.6 ± 15.3	23.0 ± 0.31a
Sukha-3 + no inoc + inorg	4.0 ± 1.1 (0.6c)	25.6 ± 1.6	146.2 ± 26.8bc	19.5 ± 4.9bc	160.0 ± 57.6	23.7 ± 0.68a
Sukha-3 + no inoc + org	6.0 ± 1.8 (0.7ab)	26.8 ± 0.4	134.9 ± 24.7c	17.3 ± 0.8c	172.3 ± 11.5	23.4 ± 0.85a
Tilki + bio-inoc + inorg	6.1 ± 0.2 (0.7ab)	28.3 ± 1.0	246.8 ± 12.0a	42.7 ± 2.1a	200.3 ± 24.3	18.5 ± 1.00b
Tilki + bio-inoc + org	6.3 ± 1.1 (0.8a)	29.6 ± 0.4	201.5 ± 49.9abc	35.3 ± 8.5abc	201.3 ± 4.1	18.94 ± 0.62b
Tilki + no inoc + inorg	3.3 ± 1.5 (0.4c)	28.6 ± 1.0	209.6 ± 22.9abc	40.6 ± 14.7ab	195.3 ± 12.3	18.5 ± 0.28b
Tilki + no inoc + org	5.5 ± 0.6 (0.75ab)	31.7 ± 4.8	219.4 ± 10.5ab	43.6 ± 1.4a	181.3 ± 23.2	18.6 ± 0.08b
*P*-value	0.50	0.81	0.06	0.40	0.67	0.51
LSD	0.19	6.31	82.71	21.43	92.33	2.42

**Notes.**

Means followed by same letter(s) do not differ significantly at 5% level of probability, *P*, Probability value, LSD, least significant difference; Bio-inoc, Rice seedling treated with *Trichoderma* spore suspension before transplanting, Non-inoc, *Trichoderma* not inoculated, the values behind ± indicates standard deviation. ^z^ values inside the parenthesis indicates the log transformed values.

The number of panicles m^−2^ was similarly and significantly affected by the different treatments. The highest number of panicles m^−2^was observed in Tilki + bio-inoculation + organic fertilization (201), followed closely by the other treatments, except for Sukhadhan-3 + bio-inoculation + inorganic fertilization treatment, which had the lowest number of panicles m^−2^ (147).

The numbers of filled grains and unfilled grains were also found to be significantly affected by the treatments, as shown in Table 3. Among all of the interacting treatment, the highest number of unfilled grains panicle^−1^ was recorded with Tilki + no inoculation + organic fertilization (43.6), which was statistically at par with Tilki + bio-inoculation + inorganic fertilization (42.7); Tilki + no inoculation + inorganic (40.6); and Tilki + bio-inoculation + organic (35.3). The lowest number was recorded with Sukhadhan-3 + no-inoculation + organic fertilization (17.3), a large drop-off.

Similarly, the number of filled grains panicle^−1^ was found to be highest also in Tilki + bio-inoculation + inorganic (246), and lowest with Sukhadhan-3 + no-inoculation + organic (135). The longest panicle length was found with Tilki + no inoculation + organic (31.7); but this was not significantly longer than with the other treatments. The lowest value for this parameter was with Sukhadhan-3 + no inoculation + inorganic (25.6) (Table 3).

These results are further analyzed and discussed in the section which follows, trying to parse and explain what patterning can be discerned by doing some rank-ordering of the treatments with regard to the respective parameters. From the comparisons made here, however, we already see indications of there being combined effects of organic fertilization and *Trichoderma* inoculation in SRI. That the highest grain yields with both varieties were obtained with *Trichoderma-* treatment and organic fertilization indicated the significant role that *Trichoderma* can play in increasing grain yield with organic SRI methods of cultivation.

## Discussion of Results through Rank-Ordering and Multiple Factor Analysis

**Figure 4:**
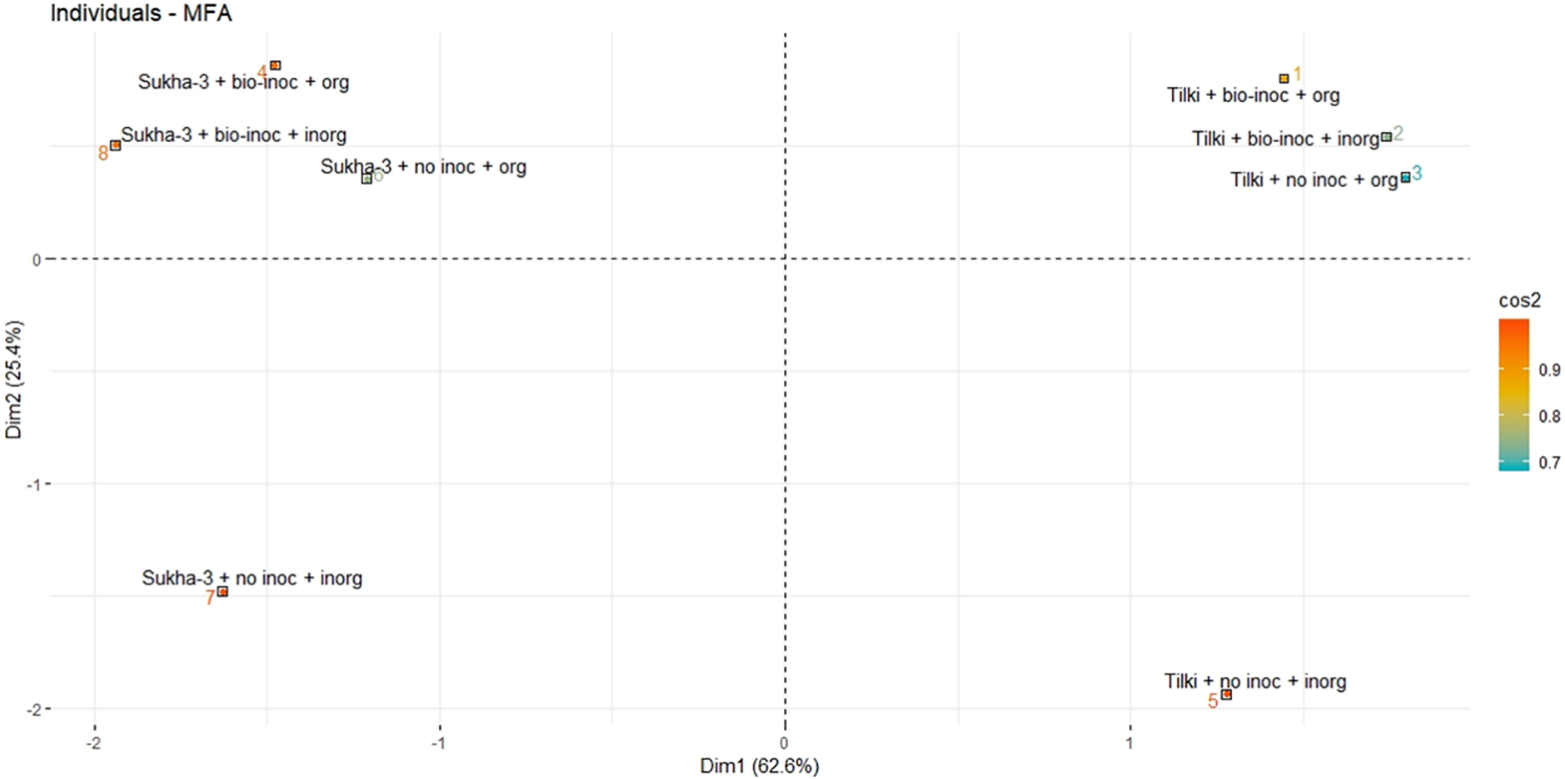
Multiple Factor Analysis of the interaction effects of *Trichoderma* inoculation under SRI management in main rice season 2015. Multiple Factor Analysis results for variable group: 1. coord, Coordinates, 2. cos2, quality of representation of interactions (treatments), 3. contrib, Contributions, 4. correlation, Correlation between groups and principal dimensions

As with any multi-factorial evaluation, presenting results and relationships can be complicated. To gain some perspective on the above findings, we rank-ordered the results reported in Table 3 and conducted Multiple Factor Analysis to calculate an average ranking for the eight different treatments according to the five positive parameters (grain yield, grain weight, panicles m^−2^, filled grains panicle^−1^, and panicle length). In Fig. 4 and Table 4, these numbers are shown with the treatments listed according to their average rank, the highest number representing the highest performance.

**Table 4:** Rank-ordering of the effects of *Trichoderma* inoculation under SRI management in main rice season 2015.

Treatments	Grain yield (mt ha^−1^)	Panicle length	Filled grains panicle^−1^	Tiller m^−2^	Test grain weight (gm)	Average rank
Tilki + bio-inoc + org	2nd	2nd	4th	1st	5th	2.8 (1st)
Tilki + bio-inoc + inorg	3rd^a^	4th	1st	2nd	7th^a^	3.4 (2nd)
Tilki + no inoc + org	5th	1st	2nd	4th	6th	3.6 (3rd)
Tilki + no inoc + inorg	7th	3rd	3rd	3rd	7th^a^	4.6 (4th)^a^
Sukha-3 + bio-inoc + org	1st	6th	5th	7th	4th	4.6 (4th)^a^
Sukha-3 + no inoc + org	4th	5th	8th	5th	3rd	5.0 (6th)
Sukha-3 + no inoc + inorg	6th	8th	6th	5th	1st	5.2 (7th)
Sukha-3 + bio-inoc + inorg	3rd^a^	7th	7th	8th	2nd	5.4 (8th)

**Notes.**

Bio-inoc Rice seedling treated with *Trichoderma* spore suspension before transplanting Non-inoc No *Trichoderma* inoculation

a Tied in rank.

Figure 4 Multiple Factor Analysis of the interaction effects of *Trichoderma* inoculation under SRI management in main rice season 2015 (Multiple Factor Analysis results for variable group: 1. coord = Coordinates, 2. cos2 = quality of representation of interactions (treatments), 3. contrib = Contributions, 4. correlation = Correlation between groups and principal dimensions) The number ranked according to the ranking of the treatments (1 is best, 7 is worst) analyzed based on five positive parameters (grain yield, grain weight, panicles m^−2^, filled grains panicle^−1^, and panicle length).

What is seen from Table 4 and Fig. 4 is that the combination of Tilki with *Trichoderma* inoculation and either organic or inorganic fertilization ranks fairly consistently at the top among the treatments. Then, fairly closely ranked in the middle range come Tilki + no inoculation + organic fertilization and Sukha-3 with either no inoculation + organic fertilization or with no inoculation and inorganic fertilization. Sukha-3 under SRI management responds roughly similarly to either combination of no *Trichoderma* inoculation plus NPK, or *Trichoderma* inoculation plus FYM. These three treatments on average are pretty similar in their results.

Interesting results were obtained from the multiple factor analysis of the interaction effects of the different treatments. Five variable groups were considered—coordinates, quality of representation of interaction of the treatments, contributions of the treatments, correlation between groups, and principal dimensions of the five positive parameters (grain yield, grain weight, panicles m^−2^, filled grains panicle^−1^, and panicle length). Each parameter represented different groups in the analysis.

We found four distinct clustering of the treatments where non-inoculated and inorganic fertilizations made for two separate clusters for different varieties at the bottom of the graph. However, treatments with both bio-inoculation and organic fertilization appeared at the top with a higher cos2 value. We observed a clear effect of bio-inoculation and organic fertilization (Fig. 4). Thus it is easy to explain the positive effect of either organic fertilization or bio-inoculation or a combination of these in both varieties. However, ranking showed greater effect of these treatments in Tilki where Tilki + bio inoculation + organic appeared frequently in the number one ranking (Fig. 4).

Then come the other three treatments that lag fairly clearly behind the others: Sukha-3 with inoculation and inorganic fertilizer, or with no inoculation but with organic fertilization, as well as Tilki that received inorganic fertilizer and no inoculation. The HYV having been bred to be responsive to inorganic fertilizer showed no response to inoculation; fertilizing it with NPK but no inoculation gave a higher ranking (3rd) than with biofertilization (6th), an interesting finding. Overall, perhaps the most important finding was that both the modern variety and the traditional one gave their best performance when receiving both inoculation and organic fertilization.

The number of panicles m^−2^ was similarly and significantly affected by the different treatments. We observed that the highest number of panicles m^−2^ was in Tilki + bio-inoculation + organic fertilization (201), followed closely by the other treatments, except for Sukhadhan-3 + bio-inoculation + inorganic fertilization treatment, which had the lowest number of panicles m^−2^ (147).

These results are consistent with the observation of Rubio et al. (2017) who when studying tomato production found higher yield and growth parameters when *T. harzianum* (T34) was applied with inorganic NPK. On the other hand, the HYV plants when inoculated with *T. harzianum* and grown with chemical fertilization gave the fewest panicles per m^2^.

These researchers concluded that NPK-fertilized tomato plants were more sensitive to salt stress than were unfertilized ones. However, it appeared that there was some synergistic effect of *Trichoderma* inoculation when combined with NPK that helped the plants to combat salt stress. When unfertilized, the tomato plants were seen to have higher growth and better tolerance of salinity when inoculated with *T. harzianum*.

The authors proposed that NPK in T34-treated plants triggered accelerated plant growth which caused some down-regulation of phytohormone networking that made plants less able to tolerate environmental stresses. The effect could be somewhat different in HYVs, however. As we noted already, Sukhadhan 3 is a drought-tolerant variety developed by IRRI with drought-tolerance QTLs (Vikram et al., 2015) that could be affected by ecosystem and environmental factors.

We saw that *Trichoderma* combined with NPK fertilization created a super-favorable environment for rice growth under SRI management, but with the genetic make-up of the drought-tolerant HYV, it could have some inherent limitations. There may be different QTLs in the traditional Tilki variety that do not inhibit utilization of nutrients, so the addition of nutrients from chemical fertilizer would augment the boost that *Trichoderma* gives rice plants under favorable conditions, resulting in higher yield from Tilki under either organic or inorganic fertilization with *Trichoderma* inoculation*.*

### General discussion of treatment effects

SRI is a strategy for modified and careful management of plants, soil, water and nutrients to provide the rice plants with the optimum growing environment. Through its alternative wetting and drying of paddy soils and by enhancing the exchange of gases in rhizosphere regions through frequent soil disturbance by a rotary weeder, SRI can be understood as improving the conditions for growth of beneficial soil microbes. Increased levels of organic matter in the soil amplify the effects of this soil and water management. Evaluations done in India and Indonesia have found that beneficial microbial populations in the rhizosphere are generally higher under SRI management (Anas et al., 2011).

*Trichoderma* is one of the fungal genera in agriculture best known for growth promotion and for biological control of plant diseases (Benítez et al., 2004; Savazzini, Longa & Pertot, 2009; Diánez Martínez et al., 2016). This genus colonizes roots and provides signaling to the plants to trigger the production of growth regulators and to induce systemic resistance to pathogens. In our study, we observed better plant architecture, higher panicle number, longer panicle length, and increased plant height in *Trichoderma*-inoculated SRI rice plants compared to plants inoculated with *Trichoderma* but grown with conventional practices. This indicated that the SRI methods of cultivation produced a more hospitable environment for *Trichoderma* than conventional rice cultivation, as has been reported also by Doni et al. (2017) from their evaluations of *Trichoderma* inoculation of rice grown with either SRI or conventional (flooded) management.

Significant increases in grain weight and yield indicated the potential for commercial application of these techniques. The better performance of rice plants inoculated with *Trichoderma* and having organic soil fertilization indicated the potency of these techniques for cultivating organically a rice crop and variety which has high market value and strong consumer demand.

Researchers have previously found higher grain yield, disease-resistance, and better plant architecture associated with *Trichoderma*-inoculated rice plants compared to non-inoculated ones. In their greenhouse experiments, (Doni et al., 2014) found plant height, leaf number, root length, root fresh weight, and tiller number to be enhanced in *Trichoderma* sp. SL2-inoculated treatments compared to non-inoculated treatment. Further, it has been seen that *Trichoderma* altered physiological processes which enabled plants to utilize resources such as water, light and nutrients more efficiently (Shoresh & Harman, 2010; Doni et al., 2014). Doni et al. (2014) have demonstrated higher photosynthetic rates, greater root length, and more water use efficiency in *Trichoderma*-inoculated plants. Further, our results are consistent with the recent work of Zaidi et al. (2017), who found in field and village-level trials in Bihar, India, that rice plants inoculated with *Trichoderma* strain S2 had higher grain yield compared to uninoculated treatments from the drought-tolerant rice variety *Sabhagi dhan*. This was found with both farmers’ practices and IRRI-recommended improved cultivation practices, with the latter giving better results.

In other reporting, Doni et al. (2015) have showed that *Trichoderma asperellum* SL2 inoculation in combination with SRI practices led to remarkably higher plant growth, germination rate, vigour index, and chlorophyll content, also prompting better plant architecture from the same genotypic potential. They concluded that SRI field management creates a more congenial environment for *Trichoderma* to contribute to plants’ growth and development traits, to more supportive soil conditions and nutrient uptake, and ultimately to the rice plants’ productivity, whereas conventional cultivation practices were seen to have detrimental effects. Doni et al. (2018) have shown further that farmers can produce and inoculate their SRI seedlings with *Trichoderma* on a cost-effective basis.

SRI has great promise to enhance yield of rice compared to conventional cultivation practice because it provides rice plants with a better growing environment regardless the variety used. In our second study, we observed no significant difference in yield between the improved dwarf variety Sukhadhan-3 and the heirloom, photoperiod-insensitive variety Tilkidhan. This result is consistent with a previous evaluation (Khadka, Acharya & Uphoff, 2014) in which, under SRI management, a two-fold increase in yield was observed from a locally-adopted rice variety known as Hansaraj Basmati in Bajang district of Nepal in the foothills of the Himalayas. Similarly, Ahmed, Dutta & Ray (2015) also found the traditional variety *Koiya Boro* under SRI management giving a 16% higher yield compared to that from currently recommended practices, and a 33% increase compared to traditional cultivation practices.

However, higher yield response is often reported for hybrid varieties under SRI management than results obtained from traditional or improved (inbred) varieties. Senthil et al. (2013) reported increased yield, reduced lodging, and faster multiplication of seed of indigenous rice varieties in Tamil Nadu state of India under system of rice intensification. They concluded that SRI methodology would be an important tool to conserve the indigenous rice varieties that are under threat of extinction as these can be produced commercially and organically under SRI management to make rice-growing more profitable and viable for farmers.

The mechanisms behind higher yield and better plant architecture as a result of inoculation with *Trichoderma* has been described by a number of researchers for various crops. Baker (1984) and Chang (1986) have described how *Trichoderma* inoculation led to growth promotion in crops such as radish, pepper, cucumber and tomato. Similarly, Harman (2000) demonstrated that *Trichoderma* inoculation leads to a proliferation of secondary roots and to increases in root length, leaf area, and seedling fresh weight. Further, Altomare et al. (1999) demonstrated a higher percentage of P and Fe in inoculated cucumber plants, with a significant increase in plant fresh weight and leaf area due to colonization of *T. asperellum* in the roots. Similarly, Borges Chagas et al. (2015) demonstrated highest rice biomass with most phosphate-solubilization efficiency in rice that had been inoculated with *T. asperelloides* and *T. harzianum*. They proposed that *Trichoderma* can enhance the availability of plant nutrients through P solubilization. Studies have also shown that *Trichoderma* spp produce growth-regulating phytohormones like auxin that stimulate plant growth and development (Contreras-Cornejo et al., 2009).

This study also evaluated the performance of two different and contrasting rice varieties under organic SRI management. We observed no significant difference in their grain yield and other parameters under either organic or inorganic fertilization when grown under SRI management. This indicated that SRI methods can match the higher-cost methods of input-dependent ‘modern’ agriculture through organic farming practices that are both less expensive and more environmentally-friendly.

Further, the very positive and productive response of an ‘unimproved’ heirloom variety when grown with organic SRI methods showed that this could even be superior to ‘improved’ varieties under optimizing crop management in terms of economic profitability. Similar results have been reported also by Debbarma et al. (2015) who observed higher grain yield and other parameters in Japonica rice under SRI management compared to conventional transplanting in organic production. We observed better grain yield when *Trichoderma* was bio-inoculated in organically-fertilized rice compared to inorganic fertilization. Plant-growth-promoting fungi such as *Trichoderma* are supported by having more access to organic matter in the soil, while inorganic fertilization can have a detrimental effect on these soil microbes, e.g., by altering the soil pH. Sharma et al. (2017) recently recorded elevated microbial counts (fungal, bacterial and actinomycetes) with organic SRI compared to both inorganic SRI and conventional transplanting methods.

## Conclusion

This study has shown the significant potential of *Trichoderma* for increasing both rice grain yield and quality, especially in combination with SRI management practices. More effect was seen with the landrace Tilki than with the HYV Sukha-5. The analysis here found the efficiency of *Trichoderma* to be greater with organic than with inorganic methods of cultivation. This effect in organic systems would be due to the more hospitable environment for *Trichoderma* build-up.

The use of *Trichoderma* inoculation as a seedling treatment can help to enhance rice production and productivity in Nepal, especially in conjunction with SRI methods and even more so with organic fertilization, which in this integrated system of rice cultivation can reduce the use and cost of chemical fertilizer. In such a system under the prevailing soil and climatic conditions, an indigenous heirloom rice variety was able to outperform in both agronomic and economic terms the more modern improved variety.

##  Supplemental Information

10.7717/peerj.5877/supp-1 Supplemental Information 1 Raw data Dry season experiment

10.7717/peerj.5877/supp-2 Supplemental Information 2 Raw data Main season experiment

## Additional Information and Declarations

### Data Availability

The following information was supplied regarding data availability: The raw data are provided in Supplemental Files.
